# Hesperidin as a Neuroprotective Agent: A Review of Animal and Clinical Evidence

**DOI:** 10.3390/molecules24030648

**Published:** 2019-02-12

**Authors:** Marziyeh Hajialyani, Mohammad Hosein Farzaei, Javier Echeverría, Seyed Mohammad Nabavi, Eugenio Uriarte, Eduardo Sobarzo-Sánchez

**Affiliations:** 1 Pharmaceutical Sciences Research Center, Kermanshah University of Medical Sciences, Kermanshah 6718874414, Iran; Marziyeh.alyani@gmail.com; 2 Medical Biology Research Center, Kermanshah University of Medical Sciences, Kermanshah 6718874414, Iran; 3 Facultad de Química y Biología, Universidad de Santiago de Chile, Casilla 40, Correo 33, Santiago 9170022, Chile; javier.echeverriam@usach.cl; 4 Applied Biotechnology Research Center, Baqiyatallah University of Medical Sciences, Tehran 1435916471, Iran; nabavi208@gmail.com; 5 Departamento de Química Orgánica, Facultad de Farmacia, Universidad de Santiago de Compostela, 15782 Santiago de Compostela, Spain; eugenio.uriarte@usc.es; 6 Instituto de Ciencias Químicas Aplicadas, Universidad Autónoma de Chile, Santiago 7500912, Chile; 7 Laboratory of Pharmaceutical Chemistry, Department of Organic Chemistry, Faculty of Pharmacy, University of Santiago de Compostela Santiago de Compostela, 15782 Santiago de Compostela, Spain; 8 Instituto de Investigación e Innovación en Salud, Facultad de Ciencias de la Salud, Universidad Central de Chile, Santiago 8330507, Chile

**Keywords:** hesperidin, neuroprotective mechanisms, Parkinson’s disease, Alzheimer’s disease, Huntington’s disease

## Abstract

Neuroprotection is the preservation of function and networks of neural tissues from damages caused by various agents, as well as neurodegenerative diseases such as Parkinson’s, Alzheimer’s, Huntington’s diseases, and multiple sclerosis. Hesperidin, a flavanone glycoside, is a natural phenolic compound with a wide range of biological effects. Mounting evidence has demonstrated that hesperidin possesses inhibitory effect against development of neurodegenerative diseases. Our review discusses neuropharmacological mechanisms for preventive and therapeutic effects of hesperidin in neurodegenerative diseases. In addition, the review examines clinical evidence confirming its neuroprotective function. Various cellular and animal models specific to neurodegenerative diseases have been conducted to evaluate the underlying neuropharmacological mechanisms of hesperidin. Neuroprotective potential of this flavonoid is mediated by improvement of neural growth factors and endogenous antioxidant defense functions, diminishing neuro-inflammatory and apoptotic pathways. Despite the various preclinical studies on the role of hesperidin in the neurodegenerative diseases, less is known about its definite effect on humans. A limited number of clinical trials showed that hesperidin-enriched dietary supplements can significantly improve cerebral blood flow, cognition, and memory performance. Further clinical trials are also required for confirming neuroprotective efficacy of this natural flavonoid and evaluating its safety profile.

## 1. Introduction

Neurodegenerative diseases are known as a group of chronic disorders characterized by a gradual loss of brain and spinal cord cells, associating with functional loss (ataxia) or sensory dysfunction (dementia), leading to eventual death and brain atrophy [1,2,3]. The progress of these disorders is accompanied by alteration of physicochemical properties in the brain and peripheral organs [4]. This group of diseases includes Alzheimer’s Diseases (AD), Parkinson’s disease (PD), Huntington’s disease (HD), Multiple Sclerosis (MS), and Amyotrophic Lateral Sclerosis (ALS) [5,6]. These diseases mostly manifest in elderly people and cause various problems, including memory dysfunction, locomotor impairment, emotional and behavioral impairments, and cognitive imperfection [7,8]. The etiology of these diseases relies on multifactorial causes, including genetic and environmental factors.

Other than these factors, brain aging is the most important cause of neurodegenerative diseases, along with the cellular and molecular factors such as potent production of oxidative stress and mutation in mitochondrial DNA, inflammatory responses, defective regulation of apoptosis, and so forth [9,10,11,12]. Most of the neurodegenerative diseases are suggested to be protein-based and accumulation and aggregation of protein in the Central Nervous System (CNS) induces oxidation and inflammation as the most significant causes of these diseases [13]. Oxidative stress, caused by overproduction of reactive oxygen species (ROS) or inadequate metabolism of the species, is believed to be a primary risk factor for neurodegeneration leading to cellular damage [2]. These species are active in the CNS and invade the post-mitotic cells, which are susceptible to being destroyed by free radicals and consequently induce progressive cellular damage in the neural system [14]. Normally, cells exert defense responses and repair mechanisms to overcome the potent oxidative stress. However, in neurodegeneration conditions like AD, the antioxidant defense system is not capable for complete counteraction of ROS-mediated effects, due to the diminished activity of antioxidant enzymes (including superoxide dismutase (SOD), catalases (CAT), and peroxiredoxins (Prxs)) [15].

Inflammation is also believed to be one of the major causes of neurodegeneration; it is defined as a condition affected by glial cells and inflammatory mediators. Inflammatory mediators, including tumor necrosis factors (TNF-α), nitric oxide (NO), inducible nitric oxide synthase (iNOS), nicotinamide adenine dinucleotide phosphate (NADPH) oxidase, and cytokines such as interleukin-1(IL-1β) play a significant role in the activation of glial cells and their neurotoxic and apoptotic effects [8,16].

The evidence of problems associated with neurodegenerative diseases stimulated neuroscientists to investigate and develop therapeutic interventions for the sake of prevention and treatment of these diseases by controlling the risk factors. Common therapies can be palliative against these dysfunctions and there is no exact medicament to halt or completely alleviate these diseases [8]. Furthermore, common therapies are associated with several side effects and there has recently been a great attention directed toward natural–based products and traditional therapies to be used as alternative or complementary therapies to common drugs [17]. Medicinal plants and their active components have long been used for enhancement of mental function. The unique properties such as antioxidant and anti-neuroinflammatory activities of plant phytochemicals [18] in association with limited side effects make them a promising agent for the management of neurodegenerative diseases. This lead us to prepare a comprehensive review on the in vivo and clinical studies to elucidate the role of hesperidin, a citrus flavonoid, for management of neurodegenerative diseases.

Due to the promising neuroprotective effects of hesperidin, different investigators dedicated their effort to explore neuropharmacological mechanisms and the molecular target of citrus flavonoids, including hesperidin. The neuroprotective effect of citrus flavonoids was comprehensively reviewed in a study conducted by Hwang et al. in 2012 [19], where the pharmacological pathways responsible for these effects were elucidated. Besides other citrus flavonoids, hesperidin was discussed in that attempt; it was reported that these natural agents can traverse blood brain barrie and can thus help the treatment of neurodegenerative diseases. According to study of Hwang et al., citrus flavonoids can exert antioxidant effect by up-regulating the expression of glutathione (GSH) peroxide, CAT, glutathione reductase, and Mn-SOD. As previously mentioned, the neuroprotective effect of hesperidin is highly dependent on the antioxidant and anti-inflammatory activities. Parhiz et al. reviewed the antioxidant and anti-inflammatory effects of citrus flavonoids [20]. Parhiz et al. concluded that apart from radical scavenging activity, increasing the antioxidant cellular defenses via a ERK/Nrf2 signaling pathway is another mechanism underlying the anti-oxidative effect of hesperidin and other citrus flavonoids. Both of these papers have reviewed the pharmacological properties of citrus flavonoids and did not focus on hesperidin.

In contrast, the present review focuses on neuroprotective effects and the hesperidin’s underlying cellular mechanisms. In a study similar to the current review, Roohbakhsh et al. [21] reviewed the neuropharmacological effect of hesperidin and hesperetin. They discussed the effect of these flavonoids on the brain diseases by reviewing some of the in vitro and in vivo experimental studies. They provided an important discussion regarding the pharmacokinetics, neuroprotective, antidepressant, antinociceptive, anticonvulsant, anxiolytic, and sedative effects of hesperidin, as well as hesperetin. Although they reviewed articles published in 2014, a large number of experimental and clinical studies (12 papers) were published since 2014, which have not been reviewed previously; our study is the first to review these papers.

Another significant factor that distinguishes Roohbakhsh and his colleagues review from the current study is that they did not mainly focused on neurodegenerative diseases. Based on the lack of a comprehensive review study on the role of hesperidin in prevention and treatment of different neurodegenerative diseases, the main focus of this study was to fill this gap. Our study provides a deep insight into the mechanisms of action and molecular targets underlying the neuroprotective activity of hesperidin, as well as the relevant clinical evidence. We have examined all the relevant databases for published studies up to 2017.

## 2. Natural Products as the Future of Neuroprotective Drugs

Common therapies are not potent in treatment and prevention of the symptomatic progression of neurodegeneration and only cause ameliorating the symptoms. Therefore, it is becoming a mandatory requirement to develop alternative medicaments for prevention and protection of these age-related diseases [22]. Natural based compounds are alternative choices, which help to control the progression of these diseases. Aromatic plants are a large group of herbal-based compounds, which have been used for a long time as neuroprotective agents. Natural polyphenols (including flavonoids, phenolic acids, vitamins, and so forth) have been found potent in several health benefits for human beings. The antioxidant and anti-inflammatory activities of these phytochemicals have been well-corroborated in several studies. Luteolin, quercetin, and fisetin possessed an anti-inflammatory effect toward neuro-inflammation by regulating IL-1 and TNF-α [23,24]. Several other flavonoids (flavonoids isolated from honey and blueberry extracts [25,26], kaempferol [27], catechin [28], naringenin [29], and acacetin [30]) potentially inhibited neuro-inflammation by controlling the pro-inflammatory biomarkers, such as nuclear factor kappa light chain enhancer of activated B cells (NF-κB) iNOS, TNF-α, NADPH oxidase, and so forth. Curcumin, resveratrol, EGb 761 (isolated from *Ginkgo biloba*), cinnamon, and various groups of plant-based compounds exhibited the potential of being intervened in neuro-inflammatory diseases [31].

Acetylcholinesterase (AChE) is an enzyme responsible for modulating the amount of acetylcholine [17]. Plant phytochemicals with anti-acetylcholinesterase activity are promising for prevention and symptomatic treatment of these diseases and improvement of cognitive function. Flavonoids isolated from green and white tea [32], as well as other flavonoids such as tiliroside and quercetin [33], terpenoids like (+)-limonene, (+)-sabinene, and trans-anethole [34] α- and β-pinene, 1,8-cineole [35,36], alkaloids (i.e., lycorine, crinine, and protopine) [36,37], and several other compounds (i.e., sterols, phenolic acids, isoflavones, steroidal alkaloids, glycosides, and xanthones) [36] exhibited inhibitory effect on AChE.

Flavonoids and their sub-classes exhibited multi-targeting abilities toward active brain sites. Some of these phytochemicals, including quercetin [38] and fisetin [39], as well as rutin [40], were found to be neuroprotective against Aβ accumulation. Further, epigallocatechin-3-gallate (EGCG, a green tea polyphenol) showed an inhibitory effect against neurotoxicity that was mediated by Aβ [37]. These compounds significantly affected the other neurodegenerative diseases (PD, HA, and ALS) [8].

According to the aforementioned reasons, medicinal plants and their phytochemicals are strongly recommended as alternative and complementary therapies for neurodegenerative diseases. Specifically, flavonoids and their sub-classes were found highly active against age-related diseases. Therefore, the present study examined the neuroprotective effect of hesperidin as one of the most active isoflavones.

## 3. Hesperidin, Chemistry, Sources, and Therapeutic Effects

**Figure 1:**
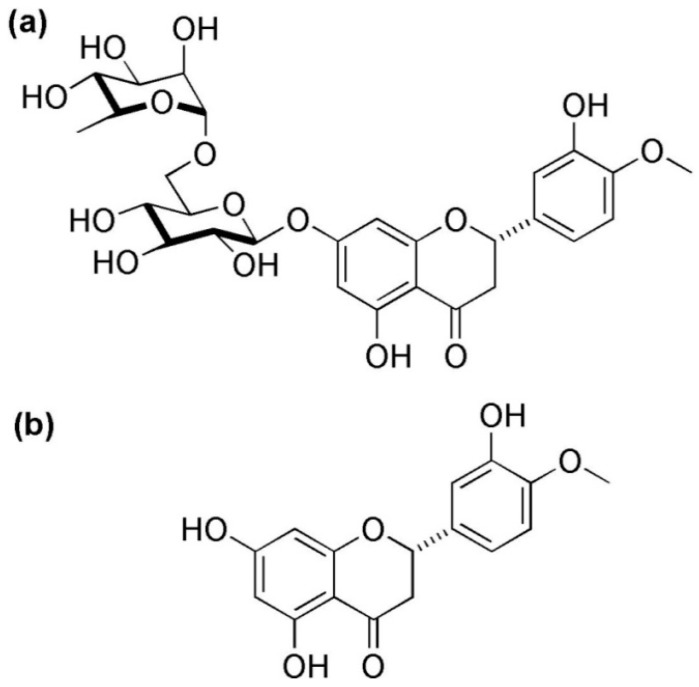
Chemical structures of hesperidin (**a**) and hesperetin (**b**).

Hesperidin (C_28_H_34_O_15_) is known as a flavanone glycoside, richly found in the citrus fruits such as lemon, sweet orange (*Citrus sinensis),* and grapefruits. The presence of this compound has also been proven in unripe sour oranges, Ponderosa lemon, *Citrus unshiu*, and *C. mitis* [7,41,42]. In addition to the Citrus species, it could be isolated from other plant genera like Fabaceae [43], Papilionaceae [7], Betulaceae [44], Lamiaceae [45], *Zanthoxylum* species *(Z. avicennae* and *Z. cuspidatum*) [40,46], and *Acanthopanax setchuenensis* [47]. Neohesperidin (as an isomer of hesperidin) is a bitter compound that is found in bitter orange (*Citrus aurantium*) [40]. Hesperidin has an aglycon (hesperitin or methyl eriodictyol) bonded to rutinose [6-*O*-(α-l-Rhamnopyranosyl)-d-glucopyranose] and/or [6-*O*-(α-l-Rhamnosyl)-d-glucose], as a disaccharide, in its structure (Figure 1) [7,47]. Thus, hesperidin (as a non-bitter flavonoid rutinoside) can be considered a β-7-rutinoside of hesperetin [7].

Various biological and pharmacological effects have been reported for hesperidin. It possesses the anti-oxidant, anti-inflammatory, and anti-carcinogenic activities [48]. Hesperidin exhibited significant mediatory effect on the extrinsic and intrinsic apoptosis of different cancerous cells [49,50,51,52]. Hesperidin and its aglycon, hesperetin, were found to be effective on different types of cancers, including gastric cancer [53], colon cancer [50], lung cancer [54,55], liver cancer [56], breast cancer [52,57,58], and prostate cancer [55]. Along with the anti-cancer activity of hesperidin, the effect of isoflavone on inflammation associated with cancer has been proven. It exhibited the inhibitory effect on the inflammatory-mediated cancers by regulating the level of inflammatory components like TNF-α, IL-1β, cyclooxygenase-2 (COX-2), and iNOS [59,60]. Hesperidin was also found to have an anti-replicative activity against some viruses [61].

The effect of hesperidin administration on blood vessel disorders such as oedema, bleeding, pleurisy, Henoch-Schonlein purpura (HSP), and tuberculosis was observed by decreasing the capillary permeability and enhancing the capillary resistance [7,62,63]. It also exerts the antihypercholesterolaemic [64], antihyperlipidaemic [65], antihypertensive, diuretic effect [66], and calcium channel blocker activity [67]. The in vivo administration of phosphorylated hesperidin caused an anti-fertility effect [68,69]. Some other biological effects, such as immuno-modulatory activity, anti-depressant, anti-allergic effect, ultra-violet protective effect, platelet, and a cell aggregation inhibitory effect, have wound healing potential and have been attributed to hesperidin [7,70]. Apart from the aforementioned biological activities, hesperidin possesses considerable neuroprotective property in various neurodegenerative diseases, such as Alzheimer’s, Parkinson’s, stroke, and Huntington’s [71,72,73,74].

One of the advantages of hesperidin–therapy is attributed to its safety, non-accumulative nature, and limited adverse effect, even during the pregnancy period. It has been administered at doses up to 5% with no mutagenic, toxic, and carcinogenic effects on mice, even after 13 weeks [7]. Furthermore, the oral administration of this phytochemical to human caused minor adverse effects in only 10% of the patients. (Placebo groups had a lower rate of 13.9% [75].) Although hesperidin is a safe phytochemical, possible interactions of this phytochemical should be considered. Coadministration of hesperidin with vincristine causes an increase in the drug uptake of this drug, in addition to hesperidin interacting with daunomycin [7].

The limited bioavailability of hesperidin and hesperetin is another significant factor which should be considered. Some solutions have been suggested to conquer this issue. For example, micronization of hesperetin causes considerable enhancement in its bioavailability and facilitated its absorption into the blood circulation [76].

## 4. Hesperidin and Neuroprotection: Mechanisms of Action Based on Animal Studies (In Vivo Studies)

### 4.1. Parkinson’s Diseases (PD)

Parkinson’s disease is one of the neurodegenerative diseases associated with the loss of dopaminergic neurons. Oxidative stress is a prominent feature in pathogenesis of PD; loss of dopaminergic neurons and the continued involvement of these features cause the appearance of non-motor symptoms of the disease (i.e., depression, mood and cognition impairment, sleep disturbance, impaired olfaction, etc.) [13,16,17]. This type of neurodegenerative disease is associated with the accumulation of ROS. The suppressive mechanisms for minimizing free radical formation and oxidative stress could involve the regulation of antioxidant enzyme levels [17,22]. In addition to oxidative stress, the oxidative metabolism of cytosolic dopamine (DA) via interaction with monoamine oxidase (MAO) enzyme plays a prominent role in the progression of PD [35].

Hesperidin, as a potent antioxidant and biomembrane stabilizer, can cause several protective effects in PD models via antioxidant and DA-enhancing mechanisms. Further, Hesperidin exhibited modulatory activity upon kappa-opioid (κ-opioid) and serotonergic 5-HT_1A_ receptors, leading to the reduction of depression symptoms [77,78]. Hesperidin was found to be effective in minimizing cognitive and depressive deficits in mice by modulating neurotransmitter systems. Moreover, it potentially inhibited the depletion of dopamine and its metabolites 3,4-dihydroxyphenylacetic acid (DOPAC) and homovanillic acid (HVA), as well as its exertion of antioxidant activity by modulating GSH levels, GPx and CAT activities, inhibiting ROS formation, and attenuating GR activity [77]. Additionally, hesperidin enhanced the locomotion efficiency, inhibited the lipid peroxidation in PD models (by decreasing malondialdehyde (MDA) content), attenuated hypercholesterolemia (by diminishing the total cholesterol and triglycerides in plasma level), and ameliorated DNA damage (by decreasing the levels of 8-hydroxydeoxyguanosine (8-OHdG)) in Chlorpyrifos-induced models of PD [79]. The neuroprotective mechanism of hesperidin in PD could also be due to regulation of other neurotransmitters, such as norepinephrine, serotonin, and epinephrine [80]. Hesperidin consumption also can be effective on PD models via down-regulating the level of proinflammatory cytokines such as TNF-α, IL-1β, IL-6, IL-4, and IL-10, as well as affecting glial fibrillary acidic protein (GFAP), iNOS, and COX-2 levels [81].

Hesperidin was found even more effective than the current drug, l-Dopa. Coadministration of hesperidin with l-Dopa increased the bioavailability of this drug in the 6-OHDA rat model of PD [80] and inhibited degeneration of cytoplasmic vacuolation mediated by 6-hydroxydopamine (6-OHDA) in the striatal and mid-brain [82]. The synergetic interaction of these two PD medicaments caused a suppressive effect on gene expressions of synuclein alpha (SNCA, as a ubiquitously expressed protein affecting the regulation of dopamine release) and Leucine-rich repeat kinase 2 (LRRK2), as an enzyme containing kinase and GTPase function, which its mutation is the most frequent genetic cause of PD. This combination also potentiated the expressions of parkin (a protein involved in the pathogenesis of autosomal recessive juvenile parkinsonism (AR-JP)) and PTEN induced putative kinase1 (PINK1, a mitochondrial kinase phosphorylates parkin) [83]. This combination of hesperidin with Sinemet (as one of the most common therapeutics for PD) decreased the side effect of this drug in Chlorpyrifos-induced PD models [79].

### 4.2. Dementia and Alzheimer’s Diseases

Alzheimer’s disease is a neurodegenerative disorder in the CNS with progressive cognitive decline and memory impairment. AD is considered the most important cause of dementia [38]. However, there is no definite etiology for AD, even though it has been well-established that its pathogenesis is closely related to cholinergic dysfunction, mitochondrial abnormalities, and oxidative stress [40]. Due to the strong memory enhancing and antioxidant effects of hesperidin, it can be considered as a potential medicament for AD and dementia.

The ability of hesperidin in increasing the anti-oxidative defense might be one of the mechanisms by which hesperidin improves cognitive function. Administration of hesperidin for 16 weeks helped improve learning and memory function by enhancing recognition index in the APPswe/PS1dE9 transgenic mouse model. It corrected the Aβ-induced mitochondrial abnormalities by reducing MDA and H_2_O_2_ levels, as well as restoring depleting GSH levels and total antioxidant capacity (T-AOC). The mitochondrial enzyme activities were also restored by elevating the mitochondrial complex I–IV enzymes activities [84]. Glycogen synthase kinase-3β (GSK-3β) is a protein kinase possesses prominent role in the mitochondrial functions and in AD. It closely affects the tau protein hyperphosphorylation and mitochondrial target [85]. Activation of this protein kinase causes increasing the oxidative damage. Hesperidin potentially rescued cognitive deficits and demonstrated mitochondrial neuroprotection effect by inhibiting restoration of this kinase. It was the possible mechanism by which hesperidin decreased the level of Aβ_1–40_ [84,86]. Hesperidin also inhibited learning and memory impairments resultant from Aluminum chloride (AlCl_3_)—induced AD, acting as an AChE inhibitor. Hesperidin attenuated amyloid precursor protein (APP) expression via NF-κB dependent pathway and suppressed the levels of Aβ_1–40_ and β- and γ-secretases (which both modulate the cleavage of APP) in the hippocampus and the cortex of the brain of rats [71]. In another study, the role of hesperidin on cognitive deterioration in rats with AD induced by AlCl_3_. AD was found to reverse the cognitive dysfunctions. By up-regulating B-cell lymphoma 2 (Bcl2) and down-regulating Bcl2-associated X proteins (Bax), expressions were included in the neuroprotective mechanism of hesperidin [87].

Beside the cognitive deficits, AD is also associated with non-cognitive and behavioral impairments. Aggregation, deposition, and neuro-inflammation of Aβ are all possible reasons for behavioral and cognitive manifestations. In addition to cognitive deficits, hesperidin was found effective in restoring the behavioral manifestation associated with AD. In a study on the APP/PS1 mice model of AD, hesperidin blocked the inflammatory process, rescued APPs production and deposition of Aβ peptides in the cortex and hippocampus of the animals, and consequently enhanced the nesting ability and social interactions of the transgenic mice [88]. The anti-inflammatory effect of hesperidin was found to be related to the mechanism, in which this herbal compound down-regulated the level of the transforming growth factor β1 (TGF-β1) immunoreactivity and NF-κB (which are known to be involved in the progression of AD) in the brain cortical region [89]. The role of TGF-β1 in stimulating the APP production and Aβ peptides deposition has been reported in a previous study conducted by Gray et al. [90].

Dementia is also a progressive dysfunction with a stepwise deterioration of memory, learning, and motor functions. The neuroprotective effect of hesperidin on the intracerebroventricular streptozotocin (ICV-STZ)-induced animal model of sporadic dementia of Alzheimer’s type (SDAT) was corroborated by testing its efficacy on spatial learning, memory, and cholinergic dysfunctions. Morris water maze escape and probe tests revealed the dose-dependent effect of hesperidin to decrease escape latency and enhance memory consolidation. The decrease of AChE activity and lipid peroxidation (by decreasing the content of thiobarbituric acid reactive substances (TBARS)), enhancement of gangliosides level, and blocking inflammatory process (by inhibiting NF-κB, COX-2, and iNOS) after administration of hesperidin exhibited the promising role of this flavonoid in alleviating symptoms of SDAT [91]. Moreover, hesperidin exerted the protective effect on vascular dementia (VD) in the rat model of l-methionine-induced hyperhomocysteinemia (HHcy). Endothelial dysfunction in addition to cognitive deteriorates, mediated by HHcy, was attenuated by hesperidin via a dose-dependent mechanism, in which nitrite and serum Hcy levels with MDA levels were decreased, AChE activity was inhibited, and the levels of GSH, SOD, and CAT were increased [92,93]. Similar results were also achieved after coadministration of hesperidin and donepezil in the scopolamine induced amnesia model, corroborating the prominent role of hesperidin in ameliorating dementia and cognitive deficits of AD [94].

### 4.3. Huntington’s Disease

Huntington’s disease is a progressive and fatal neurodegenerative disorder that is associated with a spectrum of abnormalities, such as involuntary movements, motor impairment, cognitive and memory manifestations, personality changes, neuro-psychiatric disturbances, and dementia [95,96]. A known and well-established phenotypic HD inducer in both human and animal studies is 3-Nitropropionic acid (3-NP), which was used to investigate HD neuro-psychiatric symptoms. 3-NP intoxication was accompanied by oxidative damage, body weight deficit, mitochondrial, locomotor, and grip impairments in striatum. Hesperidin treatment necessitates overcoming these impairments and enhancing locomotor and grip strength. Hesperidin’s enhancement of striatal oxidative defense and its effect on the cellular energy stores were found to be due to the modulatory effect on nitric oxide pathway [97].

To ensure the promising role of hesperidin in attenuating nitric oxide synthase expression, we compared the expressions of iNOS before and after hesperidin administration to the 3-NP-intoxicated animal models of HD. The significant role of hesperidin in suppression of iNOS level in cortical, striatal, and hippocampal regions (*p* < 0.05) corroborated its nitric oxide-related mechanism of effect on the HD models [98]. In addition to these effects, the role of hesperidin on reduction of MDA level, enhancement of CAT activity, and prevention of prepulse inhibition (PPI) of the startle response provided a strong indication that it had a beneficial role in the treatment of HD [98]. Interestingly, a microglial pathway was found to likely be involved in the protective effect of hesperidin on HD [70]. Coadministration of minocycline (as a microglial inhibitor) with hesperidin in rat models of quinolinic acid (QA) mediated HD, significantly potentiated the effect of hesperidin on excitotoxicity induced by QA. The QA-mediated apoptosis (increased level of caspase-3 activity), the QA-mediated reduction of brain-derived neurotrophic factor (BDNF, a signaling molecule secreted from activated microglia that helps to support the survival of neurons [99]) level, and the QA-mediated elevation of TNF-α level were inhibited by minocycline and hesperidin [74]. These results altogether suggest that the inhibitory effect of hesperidin on the activation of microglial cells and involvement of the microglial pathway in its neuroprotective effects against HD.

### 4.4. Multiple Sclerosis

Multiple sclerosis is a chronic and complex neuro-inflammatory demyelinating disease of the CNS, which is the major cause of neurological disability [100]. This type of neuro-inflammatory disease is commonly accompanied by axonal loss and glial scaring, in addition to the secretion of inflammatory cytokines [101]. The pathogenesis of these types of CNS disorders include the invasion and proliferation of the CD4^+^ T-cells, T-cells and macrophage infiltration, and NO production in the cerebral spinal fluid (CSF) [102,103]. The anti-inflammatory effect of flavonoids (i.e., hesperidin) and their inhibitory effect on the pro-inflammatory cytokines, together with their potential in attenuating proliferation of T-cells, makes them a promising agent in ameliorating MS. Hesperidin dose-dependently diminished demyelination in the CNS and ameliorated the clinical abnormalities in the myelin oligodendrocyte glycoprotein (MOG)-induced C57BL/6 mice model of MS. These abnormalities include excretion of pro-inflammatory cytokines such as IL-6, IL-17, IL-23, TNF-α, and Th17 cells transcription factor (ROR-γt, retinoic acid receptor-related orphan nuclear receptor gamma) and the reduction of Treg related cytokines (IL-10 and TGF-β), as well as the FoxP3 transcription factor [104].

Other than the aforementioned abnormalities, MS models demonstrated the lipid peroxidation (elevated TBARS level) and suppression of enzymatic and non-enzymatic antioxidants. Hesperidin treatment was found beneficial to alleviate these manifestations and reversed oxidative damage and histological changes of cerebral cortex caused by experimental allergic encephalomyelitis (EAE) [105]. The anti-apoptotic effect of hesperidin on the neurons of a C57BL/J6 mouse model was also corroborated via down-regulating caspase3-like immunoreactivity [105].

### 4.5. Diabetes Mellitus Associated Neurotoxicity

Diabetes is among the many independent risk factors of neurodegenerative diseases like AD and dementia [105,106,107]. Diabetes causes vascular and neurodegenerative effects on patients, leading to the fast cognitive decline; insulin resistance causes potentiating Aβ production [108]. Protein glycation and glucose autoxidation are the main reasons for damaged cell structures and disrupted cellular integrity in diabetic patients. Several studies have provided insights on the role of flavonoids as potent antioxidants with hypoglycemic and anti-inflammatory effects, in hyperglycemia of diabetes mellitus, and the incidence and progression of diabetes-induced neuro-complications [107,108,109,110,111]. Hesperidin exhibited antihyperglycemic and antidyslipidemic activities in streptozotocin induced diabetes mellitus (STZ-DM) models and successfully attenuated the overproduction of ROS by restoring the enzymatic (glutathione-S-transferase (GST) and glutathione reductase (GR); nonenzymatic endogenous antioxidants, GSH, and Nonprotein bound thiol, NP-SH). Consequently, there was also the depletion of lipid peroxidation levels (LPO) in a STZ diabetic rat brain [112]. Further, it reduces the activities of cytochrome oxidase and aldose reductase (AR), as well as sorbitol dehydrogenase (SD) [110]. The formation of xanthine oxidase (XO) in the brain of diabetic patients is included in the major pathogenesis of diabetes mellitus; hesperidin was found as one of the medicaments capable of suppressing XO levels in the diabetic brain [112]. The activity of neurotoxicity markers such as AChE and Na^+^/K^+^ ATPase were also significantly affected by hesperidin treatment. This therapeutic process possessed the ability to attenuate the diabetic neurotoxicity by controlling the sodium and potassium gradients [112,113]. These studies strongly suggest that hesperidin, as a medicament with dual anti-diabetic and AD treating attributes, is effective when targeting diabetes-induced AD.

Diabetic neuropathy (DN) is one of the most troublesome long-term complications of DM. Clinically, it can be distinguished by an increase in a nociceptive response with abnormal electrophysiological conduction [114]. The neuroprotective effect of hesperidin against STZ-induced diabetic neuropathic pain in rats has shown that hesperidin, in conjunction with insulin, reduces the diabetic condition and reverses neuropathic pain through control over hyperglycemia and hyperlipidemia, which down-regulates ROS production, releases pro-inflammatory cytokines, and elevates membrane bound enzymes [113]. The antihyperglycemic activity of hesperidin against DN is attributed to its effect on mitigating the elevated level of glycated hemoglobin (HbA1c) and overcoming the insulin resistance by increasing the level of insulin [113,115]. In the STZ-induced DN, the overproduction of TNF-α and IL-1β increase the hyperalgesia effect of polyneuropathy’s progress and maintenance [116], which could be attenuated after hesperidin treatment [113]. Furthermore, hesperidin possesses a restorative effect on normal secretome and hippocampal proteome profiles in the diabetic brain [111]. Interestingly, hesperidin was found to be a potent medicament for DN, which is highly related to both its anti-diabetic and neuroprotective effects.

The details on study design and pharmacological evidence of hesperidin against different diseases are shown in Table 1.

## 5. Clinical Evidence for Neuroprotective Potential of Hesperidin (Human Studies)

Despite the various animal-based studies on the role of hesperidin in neurodegenerative diseases, there have not been enough clinical studies devoted to the administration of hesperidin for the sake of human neuroprotection. Although a wealth of positive findings has been obtained in animal studies, the mechanism of function of this phytochemical in the human body remains to be elucidated.

In a placebo-controlled, randomized, and double-blinded clinical study, the effect of chronic administration (eight weeks) of orange juice on the cognition of 37 healthy adults (60–81 years of age) was examined. A group of adults consumed a juice with 549 mg/L hesperidin and 60mg/L narirutin (as a flavanone); another group drunk a juice with 64 mg/L hesperidin and 10 mg/L narirutin, 250 mL twice a day [117]. At the baseline there was no significant alteration in the cognitive function of these two groups, but after eight weeks the cognitive function, executive function, and episodic memory of the group that consumed orange juice with higher hesperidin content was significantly better than the group that received a lower amount of hesperidin (*p* < 0.01).

The most significant effect of hesperidin-rich juice was observed in the immediate recall (*p* = 0.006) after the follow-up period of the Consortium to Establish a Registry for Alzheimer’s disease (CERAD). Besides these effects, the chronic consumption of hesperidin-rich juice significantly decreased the diastolic blood pressure. Their study clarified that hesperidin-rich dietary interventions could prevent cognitive decline in neurodegenerative patients [117]. The underlying mechanism reflecting these effects was not clear, but other studies have claimed that flavanone consumption causes increasing cerebral blood flow (CBF) [118]. In order to examine the possibility of this mechanism, Lamport et al. carried out an acute, randomized, single-blind, placebo-controlled, clinical study on the role of citrus juice in the cognitive function and the cerebral blood flow of 44 healthy young adults (18–30). The subjects consumed 500 mL of citrus juice containing 42.15 mg hesperidin, while the control group consumed a drink containing 240 mL concentrate and 260 mL mineral water. A separate cohort of participants was chosen to examine the cerebral blood flow of functional MRI. After two hours of screening the participants, within a conscious resting state, a significant improvement was observed in the CBF of the right frontal gyrus (*p* < 0.001), as well as the Digit Symbol Substitution Test (DSST, which reflected the executive function) performance (*p* < 0.01). Although the citrus juice could enhance these parameters individually, no direct association was found between the improvement of behavioral parameters and an increase in CBF. The region-specific alteration in the CBF could be attributed to the test condition, which was carried out in the conscious resting state, in which the frontal gyrus was active [119].

In another study that focused on the role of hesperidin-rich drinks in the cognitive function of healthy middle-aged men (30–65), 5.5 g of orange pomace fiber was added to 240 mL of orange juice containing 220.46 mg hesperidin. The beverage was then administered to the treatment group. The placebo group received a drink with a similar taste and energy, but instead included a mixture of glucose, fructose, sucrose, and 0.67% citric acid in 240 mL water. Some tests corresponding to the cognitive battery were conducted to elucidate the role of drinking on the cognitive performance after a 6 h follow-up period, while only the Continuous Performance Task (CPT, which reflected the attention and executive cognitive function of participants) and finger tapping (a criterion for psychomotor speed) enhanced significantly (*p* < 0.05) 6 h post drinking hesperidin reach juice, in comparison to the placebo. The non-significant improvement of other cognitive measurements within 6 h indicated that this medication could not significantly affect global performance. On the other hand, their findings revealed the role of hesperidin-rich juice on the development of objective and subjective cognitive functions and attenuating the decline of alertness [120].

The clinical role of citrus consumption on dementia was also examined in a cohort study of 13,373 Japanese elderly participants. The incidence of dementia in participants was closely related to the consumption of citrus in a dose-response and reverse correlation. In the area where the authors carried out the clinical study, the flavonoid-rich citrus fruits were mostly consumed, which are rich in hesperidin, neohesperidin, and other flavonoids. The multivariate-adjusted hazard ratio (HR) for the incidence of dementia was found to be 0.97 for subjects with a ≤2 times/week citrus intake, while this value was 0.92 and 0.86 for subjects with a 3–4 times/week intake and almost every day citrus intake (CL = 0.95), respectivley [121]. These results indicated the protective role of citrus flavonoid in incident dementia and possibility of lowering the risk of this disorder by frequent administration of citrus fruits.

## 6. Conclusions

**Figure 2:**
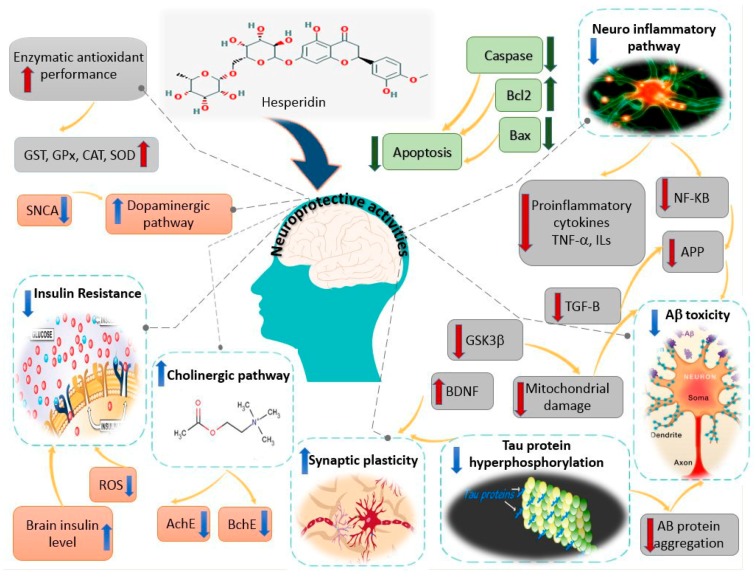
Theoretical scheme of possible molecular mechanisms underlying the neuroprotective effect of hesperidin.

Over the past decade, the underlying neuroprotective molecular mechanism of hesperidin and its metabolites has been the topic of a large number of studies. Recent studies have shown that hesperidin might have beneficial neuropharmacological effects including antidepressant, anticonvulsant, anti-inflammatory, and anticonvulsant properties, in addition to memory and locomotor enhancing. As the present review illustrates, several in vivo models specific to neurodegenerative diseases were conducted to evaluate the underlying neuropharmacological mechanisms of hesperidin. A wide range of pharmacological targets are involved in the neuroprotective potential of this flavonoid, including the improvement of neural growth factors and endogenous antioxidant defense functions, which diminish neuro-inflammatory and apoptotic pathways (Figure 2). Hesperidin can effectively protect neurons from damages induced by oxidative or nitrosative stress. Moreover, it enhances cognitive functions through various mechanisms such as elevating BDNF and reversing the disruptive effect of global cerebral I/R on memory.

Hesperidin can be considered a potential candidate for the mitigation of oxidative stress, as well as for memory impairment of the AD type. Furthermore, hesperidin showed antidepressant activities using mechanisms that differ from those of conventional antidepressant drugs. Clinical trials showed that hesperidin-enriched dietary supplements can significantly improve cerebral blood flow, cognition, and memory performance. Despite the variety of in vivo mechanistic studies on the neuroprotective activity of hesperidin, the lack of clinical trials on the therapeutic effects of hesperidin is an important limitation that can be noted, deserving further research. Moreover, less is known about the clinical aspects of this compound, such as bioavailability, the appropriate dose, tolerability, and efficacy of hesperidin and its metabolites on neurodegenerative diseases. These limitations deserve to be surmounted before expanding hesperidin treatment into humans. This can be accomplished by conducting well-designed clinical trials in patients with different types of neurodegenerative diseases. The almost published studies of the clinical efficacy of hesperidin has only been carried out on healthy participants. Studies on the role of this flavonoid, either as therapeutic or as complementary supplement, on patients with neurodegenerative diseases should be a high priority.

On the other hand, the aforementioned neuroprotective mechanisms of hesperidin can be generalized to other similar flavonoids. Furthermore, considering the versatile biological properties of hesperidin, this phytochemical may have an even a broader range of biological applications in the future. Therefore, further studies can promisingly unravel the other aspects of the therapeutic effects of hesperidin on human diseases. It is ultimately recommended that novel drug be a part of delivery systems and engineered methods for in vivo delivery of this compound to the brain.

## Figures and Tables

**Table 1:** In vivo studies demonstrating the neuroprotective effects of hesperidin.

Animal Model	Method	Mechanism of Action of Hesperidin	References
Parkinson’s Disease	6-Hydroxydopamine (6-OHDA) induced PD in rats	↑ GPx, GSH, TRAP and CAT activity ↑ DA, DOPAC, and HVA levels ↓ ROS and GR	[77]
	Chlorpyrifos—induced PD in rats	↑ CAT, GST and GSH activity ↓TG, TC, and Glucose levels ↓ MDA and SOD levels ↑ AChE	[79]
	6-Hydroxydopamine (6-OHDA) induced PD in rats	↑ DA, serotonin, epinephrine, and Norepinephrine levels	[82]
	Rotenone induced PD in rats	Suppression of SNCA gene expressions, and LRRK2 ↑ Parkin and PINK1	[83]
	1-Methyl-4-phenyl-1,2,3, 6-tetrahydropyridine (MPTP)-induced PD in mice	↓ IL-1β, TNF-α, IL- 6,4,10 ↓ GFAP, iNOS, and COX-2	[81]
Alzheimer’s Disease	APP/PS1–21 mouse model of AD	Suppression of β-amyloid deposition, APP expression, microglial activity Down-regulation of TGF-β	[88]
	APPswe/PS1dE9 transgenic mice model of AD	↓ MDA and H_2_O_2_ level ↑ GSH, T-AOC, and mitochondrial complex I–IV activity ↓ GSK-3β	[84]
	AlCl_3_ induced rat model of AD	↓ Bax and TBARS ↑ Bcl2	[84]
	AlCl_3_ induced rat model of AD	↓ AChE, APP, Aβ_1–40_, β and γ secretases levels	[71]
Sporadic dementia of Alzheimer’s type (SDAT)	Induced by intracerebroventricularstreptozotocin (ICV-STZ)	↓ AChE and TBARS ↓ NF-κB, COX-2, and iNOS ↑ Gangliosides levels	[91]
Vascular Dementia	Hyperhomocysteinemia (HHcy) induced by l-methionine in rat	↑ CAT, GSH, and SOD ↓ AChE and MDA ↓ Serum nitrite, and serum Homocysteinemia (Hcy)	[92]
Amentia	Induced by scopolamine in mice	↑ CAT and GSH ↓ AChE and TBARS	[94]
Huntington’s disease	Induced by 3-Nitropropionic acid (3-NP)	↓ NO in endothelial cells ↓ (iNOS)	[96]
		↑ CAT ↓ MDA	[97]
		Prevention of prepulse inhibition (PPI)	[98]
	Induced by quinolinic acid (QA) in rats	↓ Caspase-3 activity ↑ BDNF ↓ TNF-α	[75]
Multiple Sclerosis	Induced by myelin oligodendrocyte glycoprotein (MOG) in C57BL/6 mice	↓ IL-6, IL-17, IL-23, TNF-α, and Th17 cells transcription factor (ROR-γt) ↑ IL-10 and TGF-β	[104]
	Induced by Experimental allergic encephalomyelitis (EAE) by MOG35-55 in C57BL/J6 mouse	↓ IL-17, TNF-α, IL-1β ↓ Caspase-3 activity	[105]
Diabetes mellitus associated neurotoxicity	Induced by streptozotocin (STZ)	↑ GST, GR, GSH, and NP-SH ↓ LPO ↓ XO ↑Na^+^/K^+^ ATPase	[112]
		↑ DNA, RNA, GPX, SOD, and GSH ↓ MDA, NO, SD, ↑ Plasma glucose, Hb	[112]
Diabetic neuropathy (DN)	Induced by STZ	↓ TNF-α IL-1β ↑ HbA1c ↑ Na^+^/K^+^ ATPase	[113]

TRAP (total reactive antioxidant potential); DOPAC (3,4-dihydroxyphenylacetic acid); HVA (homovanillic acid); MDA (Malondialdyhyde); SOD (Superoxide dismutase); GST (Glutathione-*S*-transferase); TG (Triglycerides); TC (Total cholesterol); AChE (Acetyl cholinesterase); DA (Dopamine); LRKK2 (Leucine-rich repeat kinase 2); PINK 1 (PTEN induced putative kinase1); SD (sorbitol dehydrogenase).
